# Impact of ^68^Ga-PSMA-PET imaging on target volume definition and guidelines in radiation oncology - a patterns of failure analysis in patients with primary diagnosis of prostate cancer

**DOI:** 10.1186/s13014-018-0977-2

**Published:** 2018-03-01

**Authors:** K. Schiller, M. Devecka, T. Maurer, M. Eiber, J. Gschwend, M. Schwaiger, S. E. Combs, G. Habl

**Affiliations:** 1 Department of Radiation Oncology, Technical University of Munich (TUM), Munich, Germany; 2 Department of Urology, Technical University Munich (TUM), Munich, Germany; 3 Department of Nuclear Medicine, Technical University Munich (TUM), Munich, Germany; 4 Institute of Innovative Radiotherapy (iRT), Department of Radiation Sciences (DRS), Helmholtz Zentrum, Munich, Germany; 5 Deutsches Konsortium für Translationale Krebsforschung (DKTK) Partner Site Munich, Heidelberg, Germany

## Abstract

**Background:**

^68^Ga-PSMA-PET-imaging has proven to be a highly sensitive and specific diagnostic element for patients with prostate cancer (PC). Does the standard clinical target volume (CTV) cover the majority of ^68^Ga-PSMA-PET detected lymph nodes (LNs) in a primary setting?

**Methods:**

25 out of 159 patients with primary PC who underwent ^68^Ga-PSMA-PET-imaging were analyzed in the process of this study. These 25 high-risk patients had a total of 126 LNs with positive ^68^Ga-PSMA-ligand uptake. A standard CTV according to the ‘Radiation Therapy Oncology Group’ consensus was delineated and LNs were judged whether they were in- or outside of this target volume. With a Pearson correlation we additionally evaluated whether the Gleason score, the prostate-specific antigen (PSA) value or the risk according to the Roach formula correlate with a higher chance of LNs being outside of the CTV in uncommon LN locations.

**Results:**

81 (64.3%) of 126 LNs were covered by the CTV with a complete coverage of all positive LNs inside the respective radiation volume in 11 of 25 patients (44%). LNs that were *not covered* by the CTV included (para-aortic,) common-iliac, pre-sacral, obturatoric, para-rectal, para-vesical and pre-acetabular locations. In a statistical analysis neither the Gleason score, nor the PSA value, nor the calculated risk with the Roach formula correlated with LNs being inside or outside of the CTV in this patient group.

**Conclusion:**

^68^Ga-PSMA-PET-imaging proves to be a valuable asset for patients and physicians for primary diagnosis and treatment planning. In our study, trusting the RTOG consensus for CTV delineation would have led to up to 35.7% of all LNs not to be included in the clinical radiation volume, which might have resulted in insufficient radiation dose coverage.

## Zusammenfassung

**Zielsetzung:**

Die ^68^Ga-PSMA-PET Bildgebung hat in den letzten Jahren gezeigt, dass sie eine hoch-sensitive und spezifische diagnostische Möglichkeit für Patienten mit Prostatakarzinom (PK) bietet. In dieser Arbeit wird untersucht, welcher Teil der entdeckten Lymphknoten (LK), in dem nach ‘Radiation Therapy Oncology Group’ (RTOG) empfohlenem Standard “clinical target volume” (CTV) Bestrahlungsfeld, enthalten ist.

**Material & Methoden:**

Von 159 Patienten mit ^68^Ga-PSMA-PET untersuchten Patienten erfüllten 25 die Einschlusskriterien und wurden untersucht im Rahmen dieser Studie. Bei den 25 untersuchten Patienten wurden insgesamt 126 LK mittels ^68^Ga-PSMA-PET detekiert. Diese LK wurden eingeteilt ob sie innerhalb oder außerhalb des Standard Lymphabflusses nach RTOG lagen. Bei der statistischen Auswertung untersuchten wir mit Hilfe einer Pearson Korrelation ob der Gleason-score (GS), die Höhe des prostataspezifischen Antigens (PSA) oder das nach Roach kalkulierte Risiko (RR) des LK-Befalls mit der Wahrscheinlichkeit korrelierten ob entsprechende LK innerhalb oder außerhalb des CTV lagen.

**Ergebnisse:**

81 (64.3%) von 126 LK waren durch das CTV abgedeckt, bei 11 (44%) von 25 Patienten waren alle LK im CTV enthalten. Nicht abgedeckte LK waren paraaortal, im Bereich der Aa. iliacae com., präsakral, obturatorisch, pararektal, paravesikal und präacetabulär lokalisiert. Weder der GS, der PSA noch das RR korrelierten signifikant mit der Wahrscheinlichkeit, dass LK außerhalb des CTV lagen in dieser Patientengruppe.

**Schlussfolgerung:**

Die ^68^Ga-PSMA-PET-Bildgebung liefert auch in der primären Bestrahlungsplanung wertvolle Information. Bei unserer Untersuchung waren 35.7% der LK nicht im RTOG CTV abgedeckt, was potentiell zu einer insuffizienten Dosisabdeckung geführt hätte.

## Background

Finding the right treatment choice for patients with newly diagnosed prostate cancer (PC) can be challenging. If the decision is made in favor of definitive radiation therapy (RT), physicians are typically faced with the arbitration if and which lymph drainage should be covered by the RT plan. Tools such as the ‘Partin score’ or the ‘Roach formula’ to estimate the risk of lymph node (LN) involvement and recommendations by expert’s panels (e.g. the RTOG contouring atlas) respective to the radiation volume have facilitated the process [1, 2].

However, the clinical benefit of ^68^Ga-PSMA-PET imaging compared to standard clinical and histopathological factors (Gleason-Score, PSA-Level, etc.) must be analyzed in detail. It has been shown that ^68^Ga-PSMA-PET-imaging accurately detects PC lesions in a primary setting as well as for local recurrence or LN metastases [3–7].

^68^Ga-PSMA-PET-imaging has shown a major impact on staging and consecutive treatment decisions. Based on ^68^Ga-PSMA-PET-imaging in a salvage setting, RT planning was changed in more than 50% of patients and in a definitive setting in one-third of all patients, the radiation concept was altered with changes in the TNM stadium in over half of all patients [8, 9]. However, it remains vague to decide in which patient collective ^68^Ga-PSMA-PET-imaging has a benefit on the patient outcome [10]. At this point, its use is more frequently described and evidence-based in a setting of recurrence or salvage therapy planning.

In the present analysis, we evaluated the usefulness and impact of ^68^Ga-PSMA-PET-imaging from a radiation oncology perspective and analyzed whether patterns of spread based on ^68^Ga-PSMA-PET-imaging correlated with established guidelines for prostate cancer radiation treatment.

## Methods

One hundred-fifty-nine patients underwent ^68^Ga-PSMA-PET imaging for primary staging of histologically proven PC between February 2013 and September 2014. Compliance with ethical standards was met. Selection criteria for our study from this pool of patients are stated as follows:

Inclusion criteria for sub-selection of cases for this analysis were:

- Confirmed PC by biopsy
- at least one positive LN metastasis on ^68^Ga-PSMA-PET-imaging

Exclusion criteria were:

- diffuse metastatic disease
- consecutive surgical intervention by radical prostatectomy and lymphadenectomy with the histological result of pN0

Due to these criteria 25 patients were selected for further analysis and are described in this study. All patients were diagnosed with high-risk disease in accordance with the D’Amico staging system (high risk = PSA > 20 ng/ml or ≥T2c or Gleason score > 7) [11].

In seven cases an oligo-metastatic disease either to the lung, liver or bone had to be taken into considerationand could not be excluded with last certainty after staging. These patients were followed up in the respective regions by imaging but received definitive therapy to the prostate/ LNs in a curative approach and were therefore not excluded from our study population. Patients were listed in Table 1 as cM0 if metastatic disease was not confirmed. Furthermore, four patients had one or two confirmed or highly suspicious bony metastatic lesions and were individually treated in a curative approach as ‘oligo-metastasized’ patients (cM1b). These patients were treated for the respective lesions by RT with an ablative dose by means of stereotactic body RT. The entire patient criteria are included in Table 1.

**Table 1:** Patient characteristics

Characteristics	*N* = 25 (100%)
Tumor stage	
cT2b	1
cT2c	5
cT3a	1
cT3b	2
pT3a	1
pT3b	14
pT4	1
cN1	9
pN1	16
cM0	21
cM1	4
Gleason score	
6	2
7a	1
7b	2
8	5
9	12
10	2
Not available	1
*Initial PSA (ng/ml)*	
Mean (Mittelwert)	33.2
Median	15.9
Range	2–127
Not available	1
Age (years)	
Mean	68
Median	69
Range	57–80

Contrast-enhanced ^68^Ga-PSMA PET-CT imaging was either performed on a PET/CT (*n* = 15; Biograph mCT scanner, Siemens Medical Solutions, Germany) or an integrated whole-body PET/MRI system (*n* = 10, Siemens Biograph mMR, Siemens Medical Solutions, Germany) after intravenous injection of the ^68^Ga-PSMA-ligand complex. Details on imaging procedures and radiosynthesis of ^68^Ga-PSMA-HBED CC were described previously [12–14].

PET reading and interpretation was done by at least two experienced nuclear medicine physicians/ radiologists followed by a consensus interpretation. Imaging criteria for determining positive lesions were used as described previously [15]. The clinical target volume (CTV) was delineated on a planning computer tomography (CT) of one of the selected patients according to the recommendation of the ‘Radiation Therapy Oncology Group’ (RTOG) for “Pelvic Nodal Consensus CTV Contours: High Risk/ Locally Advanced Adenocarcinoma of the Prostate” [16].

This consensus stated the following as quoted and was the default for our contouring process of the CTV:

- Treatment of Presacral LNs (subaortic only)
- 7 mm around iliac vessels, carving out bowel, bladder and bone
- Commence contouring at distal common iliac vessels at L5/S1 interspace
- Stop external iliac contours at the top of femoral heads (boney landmark for inguinal ligament)
- Stop contours of obturator LNs at top of symphysis pubis

The primary target volume (PTV) was then created by adding 6 mm in all directions (CTV (+ 6 mm = PTV)). Experienced radio-oncologists reviewed all cases and were involved in delineating the LNs in the exact anatomical locations in one patient’s dataset, meaning that the anatomical relations to e.g. vessels/ musculoskeletal structures were decisive to where the LNs were delineated in the one common dataset. The LNs were contoured consistently by using a brush with 4 mm diameter and then a margin of 5 mm was applied in all directions. We then evaluated whether these LNs were covered by the CTV and/ or PTV or not. Using a color-code, LNs that would have been infield of the standard RTOG volume are depicted in dark-green and LNs that were outfield are depicted in orange (“miss”), thereby visualizing typical patterns of failure.

Statistical analysis was conducted using ‘IBM SPSS statistics’ software, version 23.0 (IBM, Armonk, USA). Pearson correlation was done to evaluate whether the Gleason score (GS), the prostate-specific antigen (PSA) value or the risk after the Roach formula correlates with a higher chance of LNs being outside of the CTV in uncommon LN locations.

## Results

**Fig. 1:**
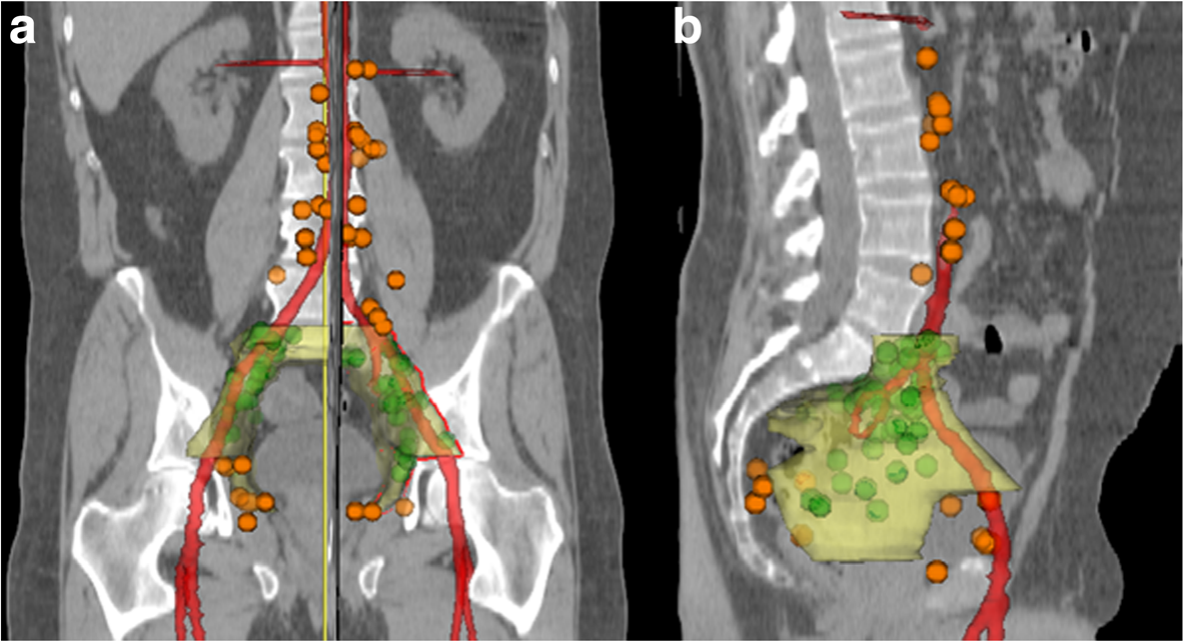
Image: Frontal (**a**) and lateral (**b**) overview depicting color-coded lymph nodes detected by PSMA-PET imaging that would have been inside (green) or outside (orange) of a standard clinical target volume (yellow) by RTOG consensus

The average age of this study population was 68 years (median 69 years, range 57–80 years). The average PSA was 33.2 ng/ml (median 15.9 ng/ml, range 2–127 ng/ml). A total of 126 ^68^Ga-PSMA-PET positive LN metastases were present in our cohort. Patients harbored between 1 and 20 positive LNs in ^68^Ga-PSMA-PET (median = 3). Eighty-one (64.3%) of 126 LNs were covered by the CTV and 90 (71.4%) by the PTV. All lesions, which showed positive ^68^Ga-PSMA-ligand uptake, were covered by the CTV in 11 patients (44%) and by the PTV (+ 6 mm margin) in 14 patients (56%). An overview of all LNs is illustrated in Fig. 1.

Analyzing the percentages of LNs covered by CTV/ PTV *without para-aortic LNs*, these increase to 76.4% (81/106 LNs) for CTV coverage and 84.9% (90/106 LNs) for PTV coverage.

**Fig. 2:**
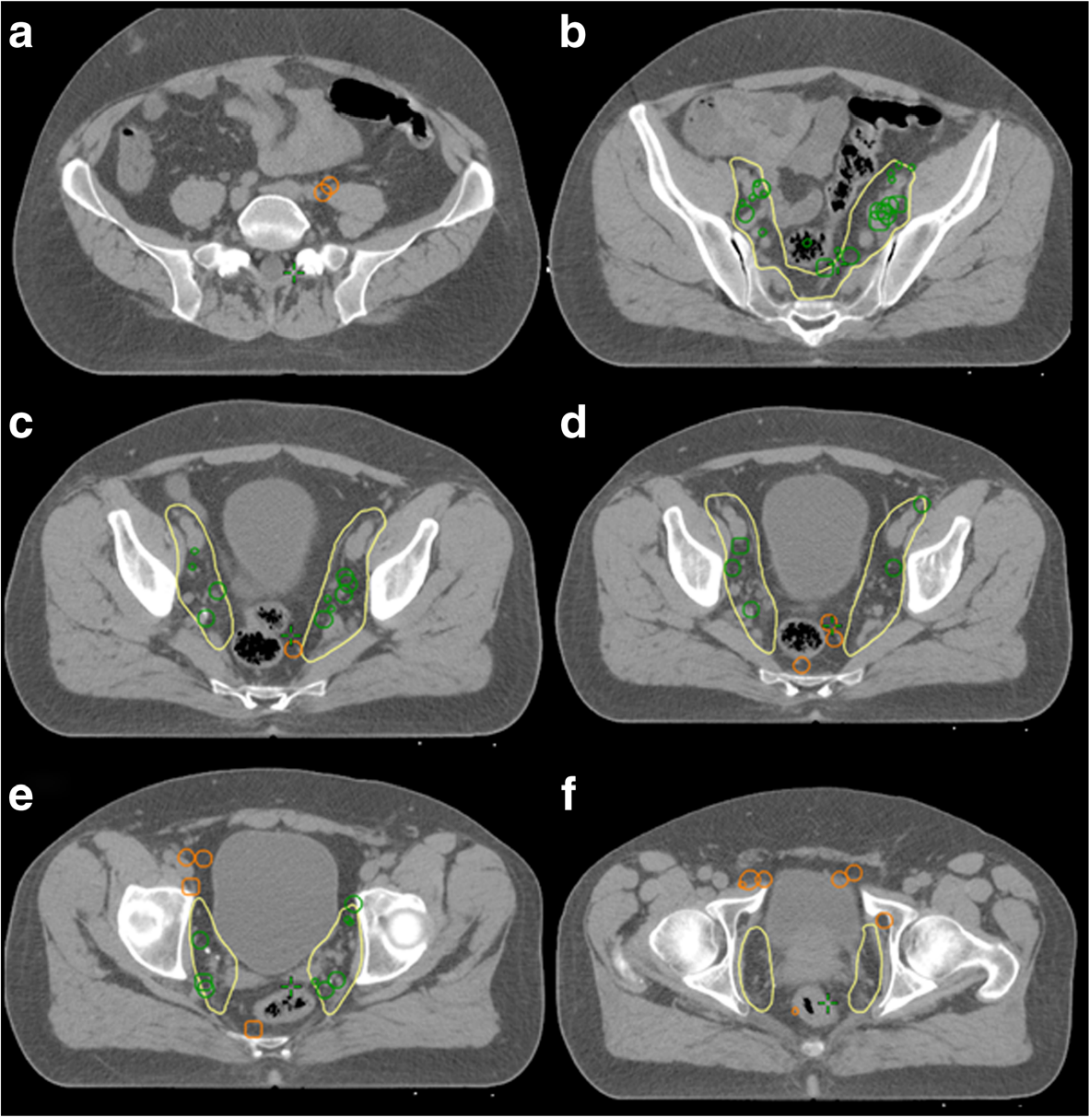
Images (**a**-**f**): Several slices of color-coded lymph nodes detected by PSMA-PET imaging that would have been inside (green) or outside (orange) of a standard radiation clinical target volume by RTOG consensus (yellow line). (**a**) lymph nodes between common iliac vessel and M. psoas, (**b**) multiple lymph nodes in the external and internal iliacal lymph drainage, (**c** + **d**) several lymph nodes para-rectal and one pre-sacral, (**e**) lymph-nodes pre-sacral, pre-acetabular and para-vesicular (**f**) more lymph nodes pararectal, pre-acetabular and para-vesicular, as well as one uncommen lymph node site lateral of the M. obturatorius internus

LNs that were *not covered* by the CTV included para-aortic, common-iliac, pre-sacral, obturatoric, para-rectal, para-vesical and pre-acetabular locations, whereupon para-aortic (*n* = 20, 15.9% of all LNs) were the most prevalent in this high-risk subgroup, followed by common-iliac (*n* = 8, 6.3%) and para-vesical (*n* = 6, 4.8%). All other locations were less frequent with a count of less than five per region. The exact location of each LN per patient are described in Table 2. Fig. 2 highlights six transversal slices with exemplary LN sites.

**Table 2:** Number and location of PET positive lymph nodes (*n* = 126) for each of the 25 patients, bold print indicates locations that would not have been covered by a standard CTV radiation field (*n* = 45) after the RTOG consensus statement

**Location/ Patient**	**1**	**2**	**3**	**4**	**5**	**6**	**7**	**8**	**9**	**10**	**11**	**12**	**13**	**14**	**15**	**16**	**17**	**18**	**19**	**20**	**21**	**22**	**23**	**24**	**25**	**∑**
**Common iliac nodes**								**1**		**2**	**1**			**2**					**1**					**1**		**8**
External iliac nodes	1	1			1	1	1			1	1	2	1	2	1			1		1	4		4	4	3	30
Internal iliac nodes	1	2	3	1				1		1		2		4	1	1	1	2	2	1	4	1		5	1	34
Obturatoric				1					1	1			1	3		1		1								9
**Obturatoric**															**1**	**1**										**2**
**Para-aortic nodes**								**2**		**1**			**1**	**5**							**1**		**4**	**3**	**3**	**20**
**Para-rectal**												**1**		**1**	**2**											**4**
**Para-vesical**	**1**							**1**		**2**													**1**		**1**	**6**
**Pre-acetabular**														**2**												**2**
Pre-sacral		4		1						1					2											8
**Pre-sacral (caudal)**								**1**		**1**				**1**												**3**
Total # of lymph-nodes	3	7	3	3	1	1	1	6	1	10	2	5	3	20	7	3	1	4	3	2	9	1	9	13	8	126

**Fig. 3:**
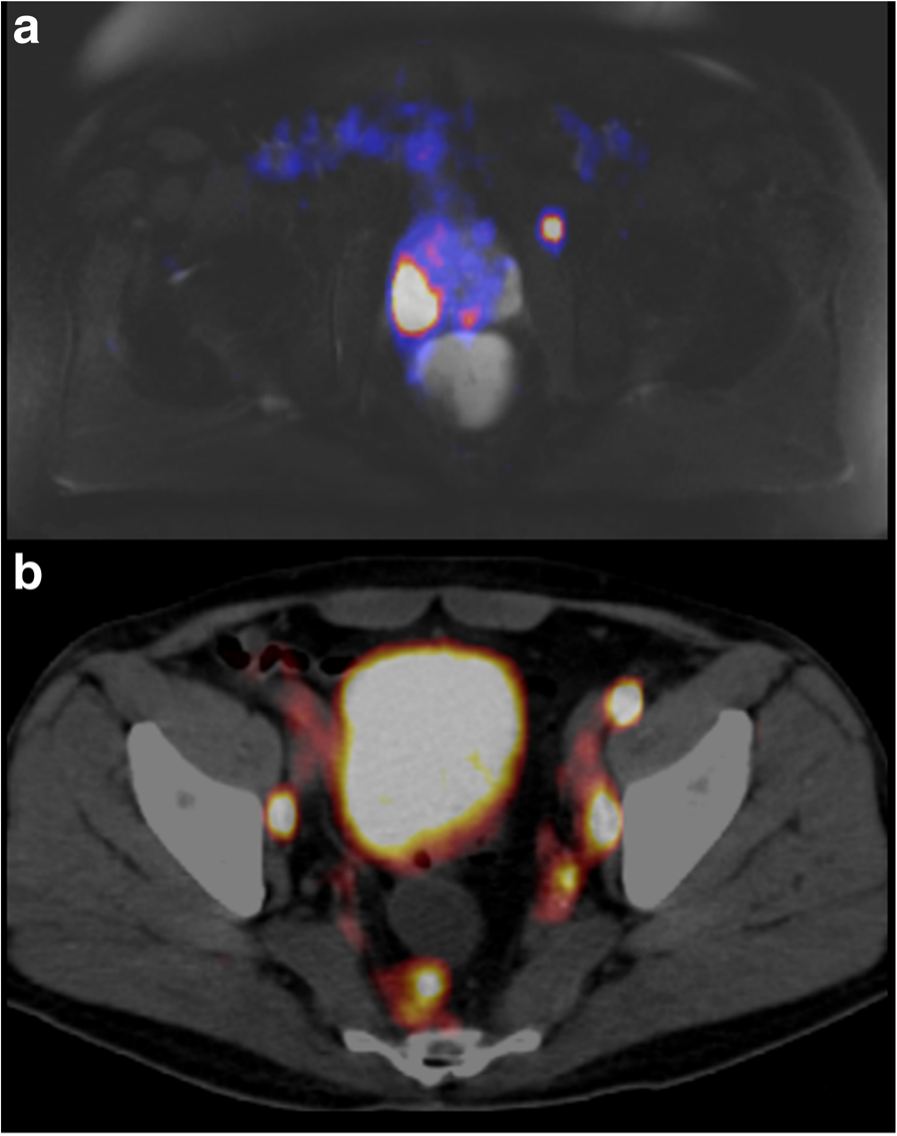
Images (**a**-**b**): lymphnodes on original ^68^Ga-PSMA-PET imaging slices: (**a**) uncommon lymphnode lateral of the M. obturatorius int. (compare image 2f) on a ^68^Ga-PSMA-PET-MRI, (**b**) several iliacal lymph nodes and one para-rectal lymph node (compare image 2d) on a ^68^Ga-PSMA-PET-CT

Two exemplary ^68^Ga-PSMA-PET-images are showing typical LN ^68^Ga-PSMA-ligand uptake; one on ^68^Ga-PSMA-PET-MRI lateral of the M. obturatorius internus (Figs. 3a and 2f) and on ^68^Ga-PSMA-PET-CT several iliacal LNs and one para-rectal LN (Images 3b and 2d).

Concerning LN sizes we also evaluated whether LNs were smaller or bigger than 1 cm in the largest diameter on CT/MRI and would therefore probably have counted as suspicious even without PSMA-ligand uptake. All together nine patients (36%) had a total of 14 LNs (14/126, 11.1%) larger than 1 cm. Six patients (24%) had only one LN, two patients (8%) had two LNs and one patient (4%) had three LNs larger than 1 cm, respectively. Out of the 14 LNs, one (7.1%) measured 4 cm in the largest diameter, three (21.4%) measured more than 1.4 cm, but less than two and the remaining ten (71.4%) LNs measured between 1 and 1.4 cm on the CT/MRI scans.

We also calculated the risk of LN involvement using the pre-treatment PSA and GS according to Roach et al. and the average risk for lymph node involvement was calculated to be 45.6% (median: 38.7%, range 6–105%).

**Fig. 4:**
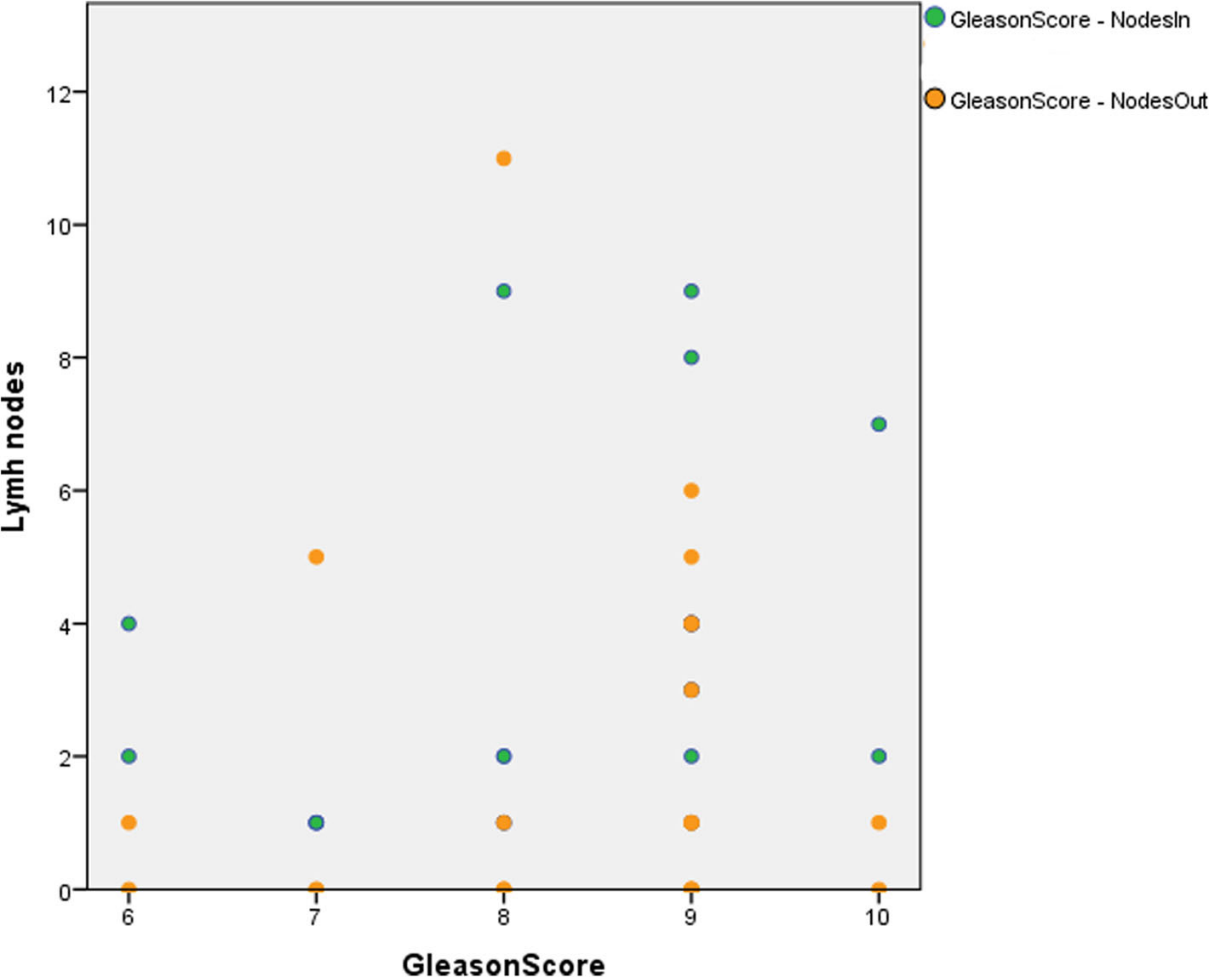
Image: Pearson correlation graph testing for connectivity between higher Gleason score and not covered lymph nodes. X-axis is Gleason score and Y-axis is the respective amount of lymph nodes. E.g. two patients had a Gleason score of ten, one of them had one lymph node outside of the CTV and the other none. Together they had nine lymph nodes inside the CTV, two and seven respectively

With a Pearson correlation, we additionally evaluated whether the GS, the PSA value or the risk according to the Roach formula correlate with a higher chance of LNs being outside of the CTV in uncommon LN locations. None of the three parameters showed a significant correlation, meaning that even with low-risk features extraordinary LN sites do occur and on the other hand, even with higher GS and/or PSA value the incidence of uncommon sites is not more frequent. Figure 4 demonstrates this by means of testing different GS and testing those for ‘covered’ and/ or ‘not covered’ LNs.

The mentioned oligo-metastases in four patients were located in the 8th and 10th thoracic vertebrae, the left pubic bone and the right pelvis.

## Discussion

Similar to our previous publication, studying the value of ^68^Ga-PSMA-PET-imaging for salvage treatment of prostate cancer, we now examined patients after primary diagnosis with no previous treatment [17]. In contrast to the postoperative situation, we expected a lower failure rate and fewer cases of suspicious LNs in extraordinary locations, as there were no previous surgical interventions in this region possibly causing changed LN drainage routes. Nevertheless, the results of this trial disagree with our expectations: 35.7% of all detected LNs would not have been covered by a standard radiation CTV, and even by the PTV 28.6% of LNs would not have been covered by a sufficient annihilating dose. That translates into over half (14/25, 56%) of the patients that potentially would have been treated inadequately without the additional information of ^68^Ga-PSMA-PET-imaging.

As for the differentiation between coverage of the CTV versus PTV, we can conclude that the LNs not included in the CTV (but in the PTV) should count as a miss, as the PTV is strictly speaking exclusively intended to account for setup errors and not adequate coverage of any clinical involvement.

Most of the LNs that would not be included in the standard CTV in our study were localized para-aortic. According to the TNM classification, these LNs are classified as M1a. Therefore, RT would not be indicated at all. On the other hand, based solely on Roach formula and without the knowledge gained by ^68^Ga-PSMA-PET-imaging, these patients would have been irradiated.

In our evaluation, eight patients showed an absolute number of 20 para-aortic LNs on ^68^Ga-PSMA-PET-imaging. Excluding these eight patients, only 16.7% (8/48) of LNs would not have been covered by the CTV. Respectively this translates into 35.3% (6/17) of the remaining patients with “missed” LNs. Hence, the recommended CTV works best for patients with exclusively regional tumor spread and further imaging investigations are indicated the higher the chance for extra-pelvic involvement. Nevertheless, this chain of thought only supports the usage of PSMA-PET-imaging in our high-risk patient population.

Looking at LN sizes, typically LNs raise suspicion on CT/ MRI if they exceed 1 cm in any diameter. It is worth pointing out that in our study group only 11.1% of LNs met that criteria. Hence the vast majority was not remarkably enlarged and was only suspected to harbor tumor cells because of PSMA-PET-imaging. With the additional information gained by ^68^Ga-PSMA-PET-imaging, former undetected LNs were included in the radiation volume to receive a sufficient dose. Often simultaneous dose escalations were applied to increase chances of tumor cell death. Data regarding the outcome and toxicity of this approach are pending and will be reported as soon as possible.

Even if our analysis did not show such a trend, a possible reason for why over one third (35.7%) of the detected LNs would not have been covered by the standard CTV is most likely due to the high-risk features in this patient subgroup; high GS might possibly be associated with an increased risk for lymphatic spread, which in turn might be reflected by PSA values.

A recent study by Sanli et al. found no statistical significance between positive and negative ^68^Ga-PSMA PET/CT findings regarding GS, which does not rule out the possibility of more aggressive patterns in terms of LNs [18].

We would like to point out that strictly following the recommendation given by the Roach formula, in three cases (12%) the LN drainage would not have been irradiated due to a risk of lymph node involvement smaller than 15% and in two further cases (8%) the risk was calculated to be between 15 and 20% per formula.

The applicability of the Roach formula as described in the original publication from 1994 in this specific high-risk population seems to be limited, as stated in the article: “The risk constraints applied to this equation are the same as those applied to the nomogram (*after Partin), where the lowest possible risk is 0%, and the highest risk is 65% (for PSAs of 40 or less)” [1, 2].

In our study, in 12% of our patient’s radiation of the lymph drainage would not have been indicated and in another 8%, the risk of involvement was calculated to be below 20%, which is the defining value for radiation to the lymphatic drainage in our clinic as the standard of care. This leads to the suspicion that the Roach formula might be *under*estimating the risk of nodal involvement. This is somewhat contradictory to results that state the Roach formula to be *over*estimating the risk of nodal involvement [19]. Keeping in mind that the patient group in this *quoted* trial had almost exclusively T1, and T2 disease and the GS was mainly 6 or 7, both statements about the Roach formula might be correct, meaning that for low-risk populations the risk might be estimated too high and vice versa. A logical consequence is to take tumor size and location (T stage) into account when deciding on treatment of lymph drainage as also discussed in the original paper of Roach et al. [1]

The Yale formula introduced in 2011 by Yu and colleagues, after evaluating 1500 patients and testing it at a collective of similar size, included the T-status ([GS - 5] × [PSA/3 + 1.5 × T]). This formula might estimate the risk, especially for patients of higher risk, more adequately but is clinically certainly not as widely used as e.g. the Roach formula up to this point [20].

Due to the retrospective nature, there are certainly limitations to this study; the collective comprises of only patients with high- or very high- risk disease. Therefore, a subgroup of our patients was already metastasized to para-aortic LNs or bony structures. Furthermore, the patients sample is small, which results inevidably in limitations concerning the statistical testing. Last, ^68^Ga-PSMA-PET-imaging itself served as the “gold standard” diagnostic tool, meaning that the detected LNs potentially also include false positives.

## Conclusion

^68^Ga-PSMA-PET-imaging proves to be a valuable asset for patients and physicians for primary diagnosis and treatment planning. In our study, trusting the RTOG consensus for CTV delineation would have led to up to 35.7% of all LNs not to be included in the clinical radiation volume, which might have resulted in insufficient radiation dose coverage.

In our analysis, in a high-risk patient group, none of the parameters examined (GS, PSA, Roach) was predictive for LNs showing enhancement in uncommon sites. This provokes the question whether ^68^Ga-PSMA-PET-imaging should be standard of care for diagnosis for patients especially with high-risk features for definitive RT.

## Abbreviations

CT: computer tomography
CTV: clinical target volume
GS: Gleason score
LN: lymph node
LNs: lymph nodes
MRI: magnet resonance imaging
PC: prostate cancer
PSA: prostate-specific antigen
PTV: primary target volume
RT: radiation therapy
RTOG: radiation therapy oncology group
